# Vascular Risk Factors and 1-Year Cognitive Change Among Individuals With Traumatic Brain Injury

**DOI:** 10.1001/jamanetworkopen.2025.25719

**Published:** 2025-08-08

**Authors:** Andrea L. C. Schneider, Katherine J. Hunzinger, Benjamin L. Brett, Danielle K. Sandsmark, Sonia Jain, Xiaoying Sun, Raquel C. Gardner, Geoffrey T. Manley, Ramon Diaz-Arrastia, Lindsay D. Nelson, and the TRACK-TBI Study Investigators, Patrick Belton, Shawn Eagle, Shankar Gopinath, Ramesh Grandhi, C. Dirk Keene, Vijay Krishnamoorthy, Christine Mac Donald, Michael McCrea, Randall Merchant, Pratik Mukherjee, Laura B. Ngwenya, David Okonkwo, Claudia Robertson, David Schnyer, Sabrina R. Taylor, John K. Yue, Ross Zafonte

**Affiliations:** 1 Department of Neurology, University of Pennsylvania Perelman School of Medicine, Philadelphia; 2 Department of Biostatistics, Epidemiology, and Informatics, University of Pennsylvania Perelman School of Medicine, Philadelphia; 3 Department of Exercise Science, Thomas Jefferson University, Philadelphia, Pennsylvania; 4 Departments of Neurosurgery and Neurology, Medical College of Wisconsin, Wauwatosa; 5 Biostatistics Research Center, Herbert Wertheim School of Public Health and Human Longevity Science at the University of California, San Diego; 6 Sagol Neuroscience Center, Sheba Medical Center, Ramat Gan, Israel; 7 Department of Neurosurgery, University of California, San Francisco, San Francisco; 8 University of Wisconsin-Madison; 9 University of Pittsburgh, Pennsylvania; 10 Baylor College of Medicine, Houston, Texas; 11 University of Utah, Salt Lake City; 12 University of Washington, Seattle; 13 Duke University, Durham, North Carolina; 14 Medical College of Wisconsin, Milwaukee; 15 Virginia Commonwealth University, Richmond; 16 University of California, San Francisco; 17 University of Cincinnati, Ohio; 18 University of Pittsburgh, Pennsylvania; 19 Baylor College of Medicine, Houston, Texas; 20 University of Texas, Austin; 21 Harvard Medical School, Boston, Massachusetts

## Abstract

**Importance:**

Traumatic brain injury (TBI) and vascular risk factors are associated with cognitive impairment, but it is unknown if individuals with vascular risk factors experience worse cognitive outcomes after TBI.

**Objective:**

To evaluate cognitive function among patients with TBI with vs without comorbid preinjury vascular risk factors.

**Design, Setting, and Participants:**

This prospective cohort study examined individuals with TBI enrolled in the Transforming Research and Clinical Knowledge in TBI (TRACK-TBI) Study between February 26, 2014, and August 8, 2018, with a 1-year follow-up. Analyses were conducted February 26, 2024, to May 29, 2025.

**Exposures:**

Vascular risk factors (ie, hypertension, diabetes, hyperlipidemia, and smoking).

**Main Outcomes and Measures:**

Participants underwent a 5-test cognitive assessment at 2 weeks, 6 months, and 1 year postinjury; tests were combined into a global cognitive factor score in primary analyses and examined individually in secondary analyses. Inverse probability of attrition–weighted generalized estimating equations adjusted for age, sex, race, ethnicity, education, TBI severity, prior TBI, and psychiatric history were used to estimate the associations.

**Results:**

The 1313 participants were a mean (SD) age of 38.7 (16.4) years, 428 were female (32.6%), 885 were male (67.4%), 393 had never smoked (29.9%), 221 had hypertension (16.8%), 98 had diabetes (7.5%), and 116 had hyperlipidemia (8.8%). Both participants with and without vascular risk factors had improvement in cognition over the first year postinjury. Compared with individuals without diabetes, participants with diabetes had lower global cognitive factor scores at 2 weeks postinjury (mean *z*-score difference, −0.25; 95% CI, −0.45 to −0.04), worse executive functioning at 2 weeks postinjury, and less improvement on processing speed over the first-year postinjury. There were no significant differences in cognitive performance comparing participants with vs without hypertension, hyperlipidemia, and smoking. Individuals with 2 or more vascular risk factors (compared with no vascular risk factors) had lower global factor score at 2 weeks postinjury (mean difference, −0.21; 95% CI, −0.19 to −0.07) and worse verbal episodic memory immediate recall, executive functioning, and processing speed at 2 weeks, but similar improvements in cognitive performance over the first-year postinjury.

**Conclusions and Relevance:**

In this cohort study of individuals with TBI, those with diabetes displayed poorer global cognitive function and executive functioning 2 weeks after TBI and recovered less in processing speed over the first-year postinjury. Given the modifiable nature of comorbid vascular risk factors, future work interrogating postinjury changes in vascular risk factor burden, over longer follow-up, is warranted.

## Key Points

**Question:**

Do individuals with traumatic brain injury (TBI) with comorbid preinjury vascular risk factors have lower cognitive function at 2 weeks postinjury and less cognitive recovery over the first-year postinjury compared with individuals with TBI without comorbid preinjury vascular risk factors?

**Findings:**

In this prospective cohort of 1313 adults with TBI, compared with participants without diabetes, participants with diabetes had worse global cognitive factor scores at 2 weeks postinjury, but similar improvement over the first-year postinjury. Hypertension, hyperlipidemia, and smoking were not associated with global cognitive performance.

**Meaning:**

These findings suggest that individuals with diabetes may have poorer global cognitive function in the short term, but had similar improvement in global cognitive function over the first year after experiencing TBI.

## Teaser

This cohort study examines cognitive function among individuals with traumatic brain injury with vs without comorbid preinjury vascular risk factors.

## Introduction

Traumatic brain injury (TBI) is often associated with significant long-lasting postinjury sequelae.^1,2^ The long-term cognitive consequences of TBI are well established, with midlife TBI being designated as 1 of 14 preventable or modifiable risk factors for dementia in the 2024 *Lancet* commission on dementia.^3^ Other preventable or modifiable midlife risk factors for later life dementia include the vascular risk factors of hypertension, diabetes, smoking, and hyperlipidemia, with hyperlipidemia having the highest population attributable fraction of these 4 individual risk factors.^3^ Furthermore, having a greater burden of these individual midlife vascular risk factors is associated with increased dementia risk compared with having fewer midlife vascular risk factors.^4^ TBI is associated with vascular and microvascular injury as well as alterations in cerebral blood flow and perfusion in the acute setting.^5^ Longer-term microvascular dysfunction that persists for years following injury has also been reported.^5^ Therefore, it is possible that individuals with comorbid preinjury vascular risk factors will experience worse post-TBI outcomes, including reduced initial cognitive recovery and greater subsequent long-term cognitive decline.

Leveraging data from the Transforming Research and Clinical Knowledge in TBI (TRACK-TBI) Study, the overall objective of this study was to examine associations of individual preinjury vascular risk factors (hypertension, diabetes, hyperlipidemia, and smoking) and cumulative vascular risk factor burden (0, 1, or 2 or more vascular risk factors) with cognitive function over the first year post-TBI. We hypothesized that individuals with vs without comorbid preinjury vascular risk factors would have a lower cognitive baseline at 2 weeks postinjury and would gain less cognitive recovery over the first year post-TBI.

## Methods

### Study Design and Population

The TRACK-TBI Study is a prospective multicenter cohort of patients with TBI who presented to and underwent a noncontrast head computed tomography scan at 1 of 18 level 1 trauma centers in the US within 24 hours of their injury between February 26, 2014, and August 8, 2018.^6^ Participants attended follow-up visits over the first year postinjury (2 weeks, 6 months, and 12-months) to assess their trajectory of recovery. Data were collected in accordance with the National Institutes of Health (NIH) National Institute of Neurological Disorders and Stroke (NINDS) TBI common data elements.^6^ The TRACK-TBI Study was approved by the institutional review board at each study site and all participants, or their legally authorized representatives, provided written informed consent. This cohort study is reported in concordance with the Strengthening the Reporting of Observational Studies in Epidemiology (STROBE) reporting guideline.

### Vascular Risk Factors

The presence or absence of preinjury vascular risk factors (hypertension, type 1 and type 2 diabetes, hyperlipidemia, and smoking) was assessed at study enrollment via self-report or proxy-report questionnaires, medical record review, and self-reported or proxy-reported medication use (eMethods in Supplement 1), as previously described.^7^ The questionnaires inquired if participants had ever received a diagnosis of hypertension, diabetes, or hyperlipidemia prior to the TBI and if participants smoked cigarettes in the 2 weeks prior to the TBI.

Our primary analyses separately examined associations of each vascular risk factor (ie, hypertension, diabetes, hyperlipidemia, and smoking) with cognition in the first-year postinjury. In secondary analyses, we considered the cumulative number of vascular risk factors (0, 1, or 2 or more vascular risk factors). In sensitivity analyses, we considered treated and untreated hypertension, diabetes, and hyperlipidemia separately.

### Cognitive Outcomes

Participants completed cognitive testing at 2 weeks, 6 months, and 1 year postinjury. The cognitive test battery included tests of verbal episodic memory (Rey Auditory Verbal Learning Test [RAVLT; higher score reflects better performance] immediate [trials 1 to 5] and delayed recall),^8^ executive functioning (Trail Making Test [TMT; lower score reflects better performance] Parts A and B),^9^ and processing speed (Wechsler Adult Intelligence Scale—Fourth Edition Processing Speed Index [WAIS-IV PSI; higher score reflects better performance]).^10^

In our primary analysis, we examined associations of vascular risk factors with a global cognitive factor score (eMethods, eFigure 1, eTable 1, and eTable 2 in Supplement 1) and in secondary analyses, we considered each cognitive test separately. In all analyses, test scores were modeled as *z*-scores standardized to the 2-week post-TBI time point.

### Statistical Analyses

Participant characteristics (overall analytic sample, stratified by number of vascular risk factors, and stratified by presence/absence of 1-year cognitive data) are shown using means and standard deviations for continuous variables and using numbers and proportions for categorical variables.

To examine the associations of each vascular risk factors with cognitive function at 2 weeks postinjury and with cognitive change over the first-year postinjury, we used inverse probability of attrition weighted generalized estimating equations (GEE) with an exchangeable covariance matrix. Each model included main estimators of individual vascular risk factor (or number of vascular risk factors), time points, and interactions between the vascular risk factor and time points, as well as the following covariates: age, sex, race, ethnicity, education, TBI severity, prior TBI, and psychiatric history (eMethods in Supplement 1). We also included interaction terms of age, sex, years of education, and TBI severity with time points as we expected these factors to be associated with the change in cognitive outcomes. Time points were included as categorical variables so that separate slopes were modeled for 2 weeks to 6 months, and 6 months to 1 year to account for potential nonlinear associations over time.

To account for attrition, weights were calculated (under the assumption of data missing at random, consistent with prior studies^11,12,13^) based on the inverse of the visit-specific probability of being observed at the visit using logistic regression, conditional on remaining uncensored in the previous visit. The model for attrition included age, sex, race, ethnicity, years of education, TBI severity, psychiatric history, prior TBI history, time point, and global cognitive factor score at the previous visit. The distribution of the weights was visually inspected, and no outliers were observed. By incorporating inverse probability of attrition weights, the results of our models are representative of the TRACK-TBI study population with cognitive data at 2 weeks.

In sensitivity analyses, we added age^2^ (and interaction term of age^2^ with time points) to our statistical model to more fully account for confounding by age. We also performed unweighted sensitivity analyses including only participants with complete cognitive data at all time points (2 weeks, 6 months, and 1 year) (ie, complete case analyses).

Statistical analyses were performed between February 26, 2024, and May 29, 2025, using R version 4.4.1 (R Project for Statistical Computing).^14^ The wgee() function in the R wgeesel package was used for the inverse probability of attrition weighted GEE and the geeglm() function in the R geepack package was used for complete case sensitivity analyses.^15,16^ Statistical significance was defined as *P* < .05, and all tests were 2-sided.

## Results

Of the 2552 adult participants aged 17 years or older in the TRACK-TBI Study, 1115 were ineligible for the present analysis due to missing data from the first cognitive assessment at 2 weeks postinjury. Of the 1437 eligible for the present analyses, 31 were excluded for missing data on vascular risk factors, 75 were excluded for missing statistical model covariates, and 18 were excluded for missing both vascular risk factor and statistical model covariate data, leaving 1313 participants included in our analytic sample (eFigure 2 in Supplement 1).

**Figure 1.**
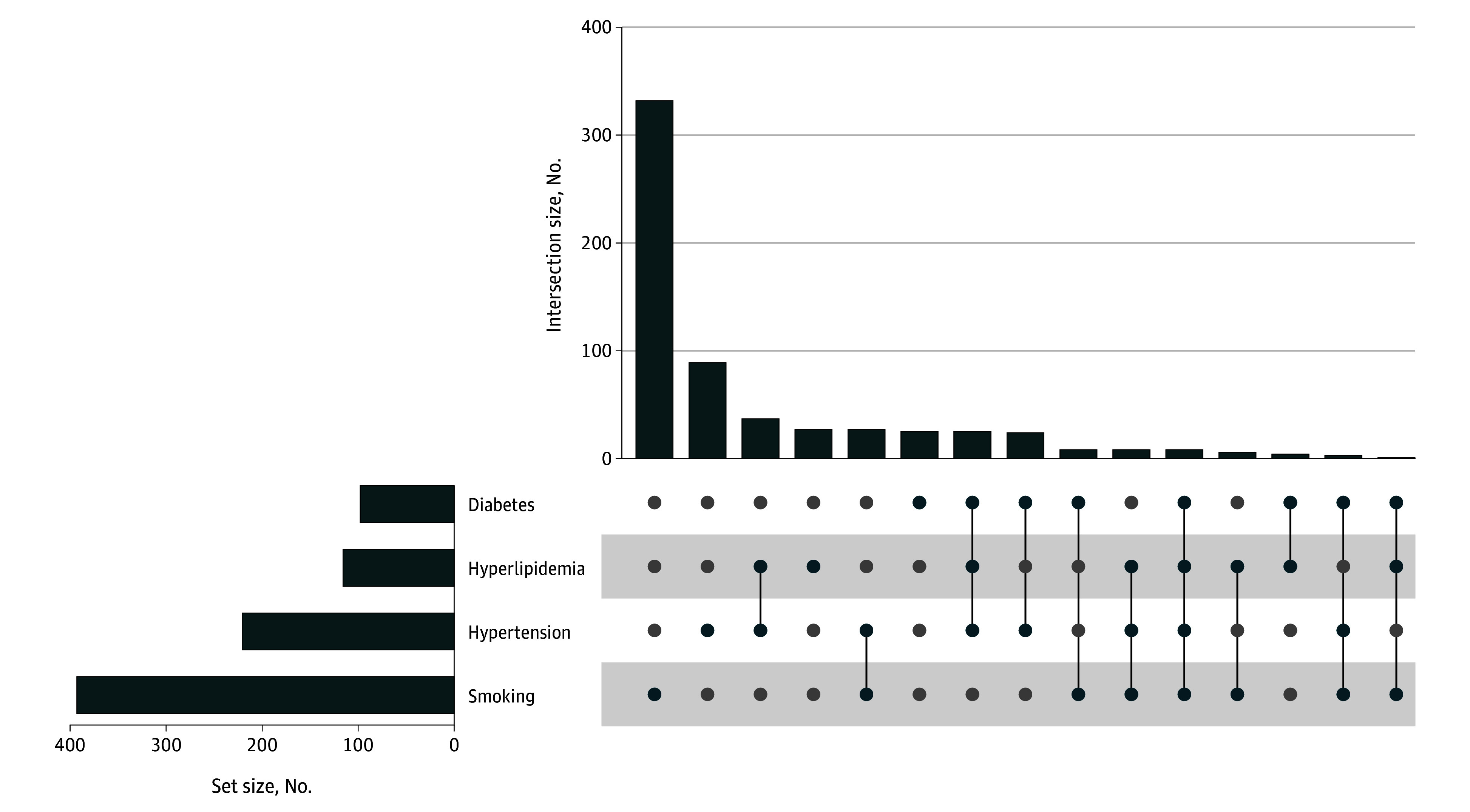
Patterns of Vascular Risk Factors in the Study Population

Overall, participants were a mean (SD) age of 38.7 (16.4) years, 428 were female (32.6%), 885 were male (67.4%), 227 were of self-reported Black race (17.3%), 995 were of self-reported White race (75.8%), 256 were of self-reported Hispanic ethnicity (19.5%), and 1057 were of self-reported non-Hispanic ethnicity (80.5%) (Table 1). Most participants (1226 [93.4%]) had a presentation of GCS 13 to 15. Among the 1226 participants with GCS 13 to 15, 404 had trauma-related intracranial findings on CT (30.8%). Smoking was the most frequently observed vascular risk factor (393 [29.9%]), with hypertension (221 [16.8%]), diabetes (98 [7.5%]), and hyperlipidemia (116 [8.8%]) being less common. The patterns of vascular risk factors in the study population are shown in Figure 1. Participants with a greater number of vascular risk factors were older compared with participants without vascular risk factors (0 vascular risk factors: mean [SD] age, 34.9 [14.0] years; 1 vascular risk factor: mean [SD] age, 38.2 [16.2] years; 2 or more vascular risk factors: mean [SD] age, 57.7 [13.4] years) (eTable 3 in Supplement 1). Among the 1313 included participants, individuals with (vs without) 1-year cognitive data were older, less educated, more likely to be of Hispanic ethnicity, have TBI of greater severity, and less likely to have hyperlipidemia and psychiatric history (eTable 4 in Supplement 1).

**Table 1.** Participant Characteristics

Characteristic	No. (%)
Overall	1313
Age, mean (SD), y	38.7 (16.4)
Sex, No. (%)	
Female	428 (32.6)
Male	885 (67.4)
Race	
Black	227 (17.3)
White	995 (75.8)
Other^a^	91 (6.9)
Ethnicity	
Hispanic	256 (19.5)
Non-Hispanic	1057 (80.5)
Education, mean (SD), y	13.7 (2.8)
TBI severity	
GCS 13 to 15 and acute head CT negative for intracranial findings	822 (62.6)
GCS 13 to 15 and acute head CT positive for intracranial findings	404 (30.8)
GCS 3 to 12	87 (6.6)
Hypertension	221 (16.8)
Treated hypertension	180 (13.7)
Untreated hypertension	41 (3.1)
Diabetes	98 (7.5)
Treated diabetes	72 (5.5)
Untreated diabetes	26 (2.0)
Hyperlipidemia	116 (8.8)
Treated hyperlipidemia	92 (7.0)
Untreated hyperlipidemia	24 (1.8)
Smoking	393 (29.9)
Prior TBI	291 (22.2)
Psychiatric history	294 (22.4)
Cognitive test scores at 2 wk post-TBI, mean (SD)	
Global cognitive factor, *z *score	0.05 (0.9)
RAVLT trials 1-5 total (immediate recall)	46.1 (10.3)
RAVLT delayed recall	8.8 (3.4)
TMT part A, s	32.0 (16.3)
TMT part B, s	82.4 (51.4)
WAIS-IV PSI	95.2 (15.7)

Abbreviations: CT, computed tomography; GCS, Glasgow Coma Score; RAVLT, Rey Auditory Verbal Learning Test; TBI, traumatic brain injury; TMT, Trail Making Test; WAIS-IV PSI, Wechsler Adult Intelligence Scale—Fourth Edition Processing Speed Index.

^a^ Other race includes American Indian, Alaskan Native, Asian, Native Hawaiian or Pacific Islander, and unknown.

**Figure 2.**
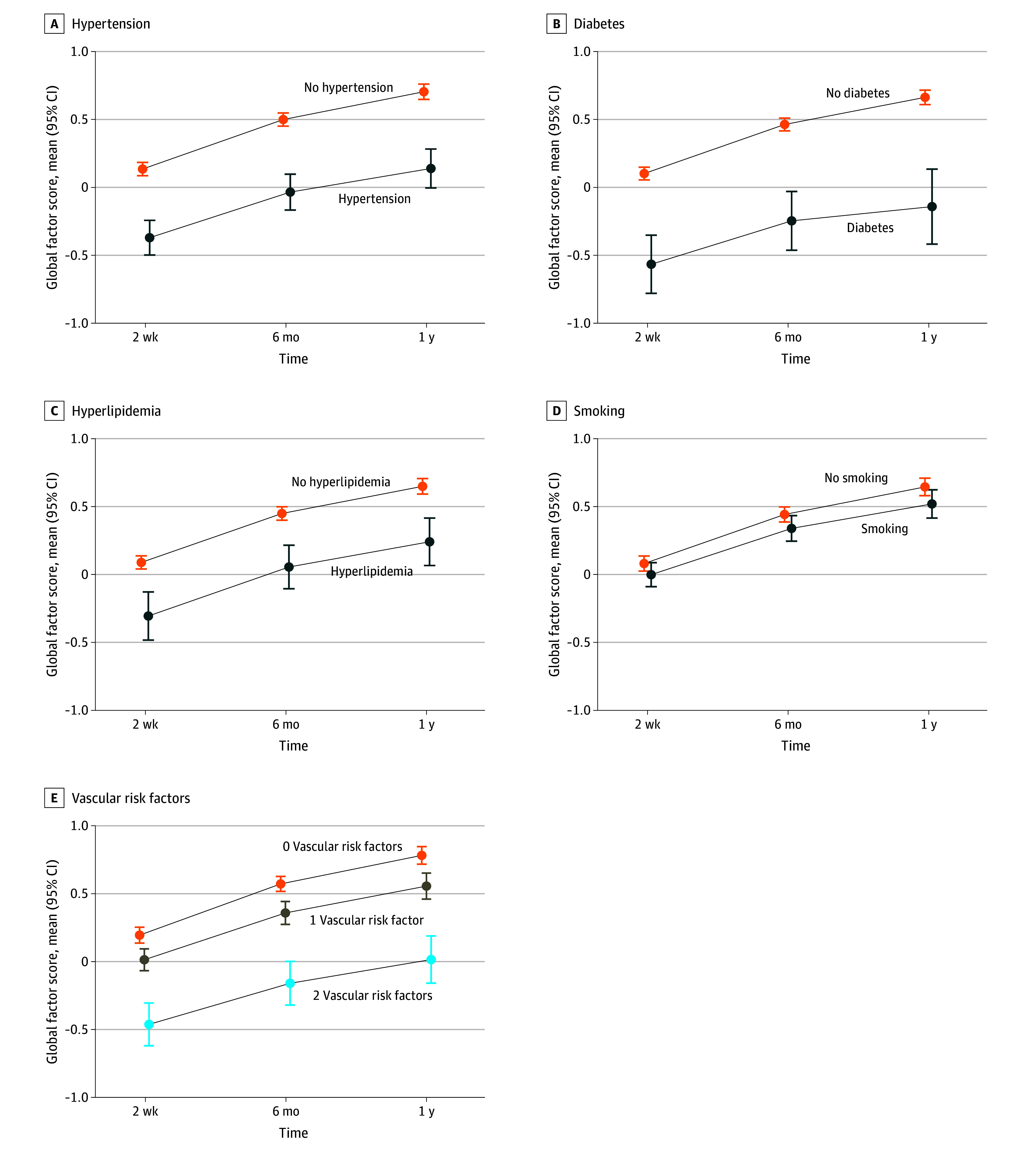
Mean (95% CI) for Cognitive Performance (Global Cognitive Factor Score) at 2 Weeks, 6 Months, and 1 Year Postinjury by Vascular Risk Factor Status

Both participants with and without vascular risk factors experienced improvement in cognition over the first-year postinjury; the unadjusted means and 95% CIs for the global cognitive factor score at 2 weeks, 6 months, and 1 year postinjury are shown stratified by vascular risk factor status in Figure 2. For individual vascular risk factors, the 2-week cognitive factor score was significantly lower among individuals with vs without hypertension (mean, −0.37; 95% CI, −0.49 to −0.24 vs mean, 0.14; 95% CI, 0.09 to 0.19), diabetes (mean, −0.56; 95% CI, −0.78 to −0.35 vs mean, 0.10; 95% CI, 0.06 to 0.15), and hyperlipidemia (mean, −0.31; 95% CI, −0.48 to −0.13 vs mean, 0.09; 95% CI, 0.04 to 0.14), whereas the 2-week cognitive factor score was similar by smoking status. By number of vascular risk factors, individuals with 2 or more vascular risk factors had the lowest 2-week cognitive factor score (mean, −0.46; 95% CI, −0.62 to −0.30), followed by individual with 1 vascular risk factor (mean, 0.01; 95% CI, −0.07 to 0.09), with individuals with no vascular risk factors having the highest 2-week cognitive factor score (mean, 0.19; 95% CI, 0.14 to 0.25).

After adjustment for confounders, participants with diabetes had lower global cognitive factor scores at 2 weeks postinjury compared with participants without diabetes (mean difference, −0.25; 95% CI, −0.45 to −0.04) (Table 2). Participants with each individual vascular risk factor had similar improvement in the global cognitive factor score over the first-year postinjury compared with participants with no vascular risk factors. In analyses of vascular risk factor burden, individuals with 2 or more vascular risk factors (compared with no vascular risk factors) had lower global factor score at 2 weeks post injury (mean difference, −0.21; 95% CI, −0.19 to −0.07) (Table 3). Participants with no vascular risk factors, 1 vascular risk factor, and 2 or more vascular risk factors all had similar improvement in global cognitive factor score over the first-year postinjury.

**Table 2.** Associations of Preinjury Vascular Risk Factors With Cognition at 2 Weeks and Change in Cognition From 2 Weeks to 1 Year Postinjury^a^

Model	Risk factor, mean (95% CI)			
	Hypertension vs no hypertension	Diabetes vs no diabetes	Hyperlipidemia vs no hyperlipidemia	Smoking vs no smoking
**Global cognitive factor, *z* score**				
Difference at 2 wk	−0.06 (−0.18 to 0.06)	−0.25 (−0.45 to −0.04)^b^	−0.11 (−0.27 to 0.06)	−0.07 (−0.16 to 0.01)
Difference in change from 2 wk to 1 y	0.01 (−0.12 to 0.14)	−0.18 (−0.40 to 0.04)	0.10 (−0.04 to 0.23)	−0.01 (−0.10 to 0.08)
**RAVLT trials 1-5 total (immediate recall), *z* score **				
Difference at 2 wk, (95% CI)	−0.11 (−0.27 to 0.04)	−0.05 (−0.26 to 0.15)	−0.12 (−0.30 to 0.06)	−0.09 (−0.20 to 0.02)
Difference in change from 2 wk to 1 y	−0.12 (−0.30 to 0.07)	−0.18 (−0.42 to 0.05)	−0.06 (−0.26 to 0.15)	−0.11 (−0.25 to 0.04)
**RAVLT delayed recall, *z* score **				
Difference at 2 wk	−0.09 (−0.26 to 0.07)	0.05 (−0.17 to 0.27)	−0.03 (−0.23 to 0.17)	−0.05 (−0.17 to 0.07)
Difference in change from 2 wk to 1 y, (95% CI)	−0.11 (−0.30 to 0.08)	−0.16 (−0.41 to 0.09)	−0.07 (−0.28 to 0.14)	−0.12 (−0.26 to 0.03)
**TMT part A, *z* score **				
Difference at 2 wk, (95% CI)	0.07 (−0.09 to 0.23)	0.28 (0.02 to 0.54)^b^	0.07 (−0.14 to 0.28)	0.05 (−0.06 to 0.16)
Difference in change from 2 wk to 1 y, (95% CI)	−0.03 (−0.23 to 0.16)	0.09 (−0.25 to 0.43)	−0.10 (−0.31 to 0.11)	0.02 (−0.10 to 0.15)
**TMT part B, *z* score**				
Difference at 2 wk, (95% CI)	−0.01 (−0.17 to 0.15)	0.30 (0.04 to 0.56)^b^	0.09 (−0.11 to 0.30)	0.07 (−0.03 to 0.18)
Difference in change from 2 wk to 1 y, (95% CI)	−0.04 (−0.25 to 0.17)	0.35 (−0.01 to 0.72)	−0.21 (−0.44 to 0.02)	0.00 (−0.13 to 0.13)
**WAIS-IV PSI, *z* score**				
Difference at 2 wk, (95% CI)	−0.02 (−0.17 to 0.13)	−0.18 (−0.38 to 0.01)	−0.14 (−0.32 to 0.04)	−0.06 (−0.17 to 0.05)
Difference in change from 2 wk to 1 y, (95% CI)	−0.05 (−0.21 to 0.11)	−0.26 (−0.50 to −0.02)^b^	0.06 (−0.11 to 0.23)	−0.04 (−0.16 to 0.09)

Abbreviations: GEE, generalized estimating equations; IPA, inverse probability of attrition; RAVLT, Rey Auditory Verbal Learning Test; TBI, traumatic brain injury; TMT, Trail Making Test; WAIS- IV PSI, Wechsler Adult Intelligence Scale—Fourth Edition Processing Speed Index.

^a^ IPA weights-adjusted GEE models were performed to assess the association of each individual vascular risk factor with each cognitive outcome. Each model included individual vascular risk factor, time points, age, sex, race, ethnicity, years of education, TBI severity, prior TBI and psychiatric history, and the following interactions (vascular risk factor × time points, age × time points, sex × time points, years of education × time points, TBI severity × time points).

^b^ *P* < .05.

**Table 3.** Associations of the Number of Preinjury Vascular Risk Factors With 2-Week Cognition and Change in Cognition From 2 Weeks to 1 Year Postinjury

Model	Factor, mean (95% CI)	
	1 vs 0 Vascular risk factor	≥2 vs 0 Vascular risk factors
**Global cognitive factor, *z* score**		
Difference at 2 wk	−0.03 (−0.11 to 0.05)	−0.21 (−0.19 to −0.07)^b^
Difference in change from 2 wk to 1 y	−0.02 (−0.10 to 0.07)	−0.04 (−0.20 to 0.12)
**RAVLT trials 1-5 total (immediate recall), *z* score**		
Difference at 2 wk	−0.11 (−0.21 to 0)	−0.18 (−0.35 to 0)^b^
Difference in change from 2 wk to 1 y	−0.12 (−0.26 to 0.02)	−0.18 (−0.39 to 0.04)
**RAVLT delayed recall, *z* score**		
Difference at 2 wk	−0.06 (−0.17 to 0.06)	−0.08 (−0.35 to 0.11)
Difference in change from 2 wk to 1 y	−0.16 (−0.30 to −0.02)^b^	−0.17 (−0.39 to 0.05)
**TMT part A, *z* score**		
Difference at 2 wk	0.00 (−0.10 to 0.10)	0.23 (0.03 to 0.43)^b^
Difference in change from 2 wk to 1 y	0.04 (−0.08 to 0.15)	0.01 (−0.24 to 0.25)
**TMT part B, *z* score**		
Difference at 2 wk, (95% CI)	0.03 (−0.07 to 0.13)	0.16 (−0.04 to 0.35)
Difference in change from 2 wk to 1 y	0.00 (−0.13 to 0.12)	0.04 (−0.22 to 0.29)
**WAIS-IV PSI, *z* score**		
Difference at 2 wk	0.05 (−0.06 to 0.15)	−0.22 (−0.38 to −0.05)^b^
Difference in change from 2 wk to 1 y	−0.05 (−0.17 to 0.07)	−0.10 (−0.28 to 0.08)

Abbreviations: GEE, generalized estimating equations; IPA, inverse probability of attrition; RAVLT, Rey Auditory Verbal Learning Test; TBI, traumatic brain injury; TMT, Trail Making Test; WAIS-IV PSI, Wechsler Adult Intelligence Scale—Fourth Edition Processing Speed Index.

^a^ IPA weights-adjusted GEE models were performed to assess the association of number of vascular risk factors with each cognitive outcome. Each model included number of vascular risk factors, time points, age, sex, race, ethnicity, years of education, TBI severity, prior TBI, and psychiatric history, and the following interactions (number of vascular risk factors × time points, age × time points, sex × time points, years of education × time points, TBI severity × time points).

^b^ *P* < .05.

In secondary analyses evaluating each cognitive test separately, the adjusted 2-week postinjury TMT Parts A and B *z*-scores were higher (indicative of worse performance) among participants with vs without diabetes (mean difference TMT Part A, 0.28; 95% CI, 0.02 to 0.54 and mean difference TMT Part B, 0.30; 95% CI, 0.04 to 0.56) (Table 2); the adjusted 2-week *z*-scores on other tests were similar by diabetes status. The adjusted 2-week *z*-scores on all individual cognitive tests were similar by hypertension, hyperlipidemia, and smoking status. The 1-year improvement on all tests was similar among individuals with and without hypertension, hyperlipidemia, and smoking. Individuals with diabetes had less improvement over 1 year on the WAIS-IV PSI compared with individuals without diabetes (mean difference in change, −0.26; 95% CI, −0.50 to −0.02), but had similar 1-year improvement on other individual cognitive tests. By number of vascular risk factors, individuals with 1 vascular risk factor had similar adjusted 2-week cognitive scores on all tests (Table 3). Individuals with 2 or more vascular risk factors had worse adjusted 2-week *z*-score on the RAVLT immediate recall TMT part A and on the WAIS-IV PSI. Individuals with no vascular risk factors, 1 vascular risk factor, and 2 or more vascular risk factors had similar improvement on all tests over the first-year postinjury, with the exception that individuals with 1 vascular risk factor improved slightly less compared with individuals with no vascular risk factors on the RAVLT delayed recall (mean difference in change, −0.16; 95% CI, −0.30 to −0.02); this effect size was similar to that of the 2 or more vascular risk factor group, which had lower precision.

In sensitivity analyses considering treated and untreated hypertension, diabetes, and hyperlipidemia separately, participants with treated diabetes had lower global cognitive factor score at 2 weeks postinjury compared with participants without diabetes (mean difference, −0.32; 95% CI, −0.57 to −0.07) and less improvement over 1 year (mean difference in change, −0.29; 95% CI, −0.57 to −0.02) (eTable 5 in Supplement 1). Participants with treated diabetes vs without diabetes also had worse 2-week score on TMT Parts A and B, and less improvement over 1 year on the TMT Part B and WAIS-IV PSI. Global cognitive factor scores at 2 weeks and over the first-year postinjury were similar for treated and untreated hypertension and hyperlipidemia and for untreated diabetes compared with individuals without each respective vascular risk factor.

In sensitivity analyses adding age^2^ as a covariate, results were of similar or slightly attenuated magnitude (eTable 6 and eTable 7 in Supplement 1). Similarly, in our unweighted complete case sensitivity analysis, results were of similar or slightly attenuated magnitude (eTable 8 and eTable 9 in Supplement 1).

## Discussion

Our primary analyses found that individuals with comorbid diabetes had worse global cognitive performance at 2 weeks post-TBI (first assessment postinjury), but that global cognitive improvement throughout the first year postinjury was similar among individuals with vs without vascular risk factors (including hypertension, diabetes, hyperlipidemia, and smoking). Our secondary analyses suggested that individuals with comorbid diabetes had worse executive functioning at 2 weeks postinjury and that there was less improvement in processing speed among individuals with diabetes throughout the first-year postinjury. Individuals with vs without hypertension, hyperlipidemia, and smoking performed similarly over the first-year postinjury on individual cognitive tests. Future work is warranted to evaluate the association of comorbid preinjury vascular risk factors, particularly diabetes, with longer-term cognitive outcomes and to investigate the impact of the development of comorbid vascular risk factors in the postinjury time period on long-term cognitive trajectories as modification of vascular risk factors is a viable avenue to influence cognitive trajectories.

A large body of literature demonstrates associations of midlife vascular risk factors with later life cognitive outcomes and dementia risk.^3^ There has been less robust study of associations of vascular health with cognitive change after TBI despite several prior studies providing evidence of higher prevalence of vascular risk factors and cardiovascular disease among individuals with vs without TBI.^17,18,19,20,21^ A prior study of US veterans reported an additive association between TBI and cardiovascular disease with dementia risk during a mean of 7 years of follow-up.^22^ Our study builds upon this prior literature by providing evidence that individuals with comorbid vascular risk factors (namely diabetes) at the time of injury have lower cognition at 2 weeks postinjury and that individuals with diabetes have slower recovery of processing speed over the first-year postinjury. These observed statistically significant effect sizes for diabetes were small, but are clinically meaningful and consistent with prior studies which report impairment in processing speed among individuals with diabetes.^23,24^ These stronger associations of diabetes (compared with other vascular risk factors) with cognitive function postinjury appear to be driven by the subset of individuals with diabetes who are treated with medications, which is likely an indicator of increased diabetes severity or duration. No associations were seen for hypertension, hyperlipidemia, or smoking with cognitive change in our cohort, which may be associated with the relatively younger age of included participants as prior studies in late-midlife-to-older aged cohorts have shown consistent associations of these factors with cognition^3,25^; further work among older individuals is warranted.

We observed associations of the number of comorbid preinjury vascular risk factors with 2-week cognitive test scores globally, and in memory immediate recall and processing speed, whereby 2 or more vascular risk factors were associated with lower 2-week score compared with no vascular risk factors. Change in cognition over the first-year postinjury was similar by number of comorbid vascular risk factors. The lower prevalence of most of the vascular risk factors in our younger cohort (hypertension, diabetes, and hyperlipidemia all had less than 20% prevalence) may explain these findings. In addition, the lower prevalence of vascular risk factors in our cohort meant that we were unable to investigate associations between different combinations of vascular risk factors with cognitive recovery over time. Further study in older cohorts where vascular risk factors are more common is warranted to investigate how specific combinations of vascular risk factors may impact post-TBI cognitive recovery trajectories during longer periods of follow-up.

### Limitations

This study has limitations. First, although we investigated associations between preinjury vascular risk factors and cognitive change over the first-year postinjury, changes in modifiable vascular risk factor status may have occurred after injury which could have contributed to cognitive recovery. In the TRACK-TBI Study, vascular risk factors were only ascertained at study enrollment; as such, we were unable to evaluate vascular risk factors as time-varying exposures in our analyses. Furthermore we did not have information on disease duration or severity for each of the vascular risk factors, but we were able to evaluate associations of treated and untreated vascular risk factors with cognitive outcomes. Future studies should investigate associations of both preinjury and postinjury vascular risk factor status (with consideration of severity) with cognition in order to inform if modification of vascular risk factors may have the potential to improve cognitive outcomes after TBI. Second, as with many studies in TBI populations,^26^ the TRACK-TBI Study has had study attrition over time with resultant missing 1-year cognitive outcome data in 32.7% of participants. To address loss-to-follow-up over study duration, we incorporated inverse probability of attrition weights^27^ so that the results of our models are representative of the TRACK-TBI Study population with cognitive data at 2 weeks. The methods we used (ie, inverse probability of attrition weighting) operate under the assumption that data are missing at random and it is possible that the probability of missingness may depend on cognitive function (ie, unobserved data) at 6 months and 1 year. However, as our eligible population was required to have cognitive data at 2 weeks postinjury, we do have some information on the cognitive status of participants with missing 6-month or 1-year cognitive data, and our weights were calculated from an attrition model where the corresponding cognitive score at the prior visit was considered. Third, the TRACK-TBI Study population consists of individuals with a TBI event that necessitated care at a level 1 trauma center so our results may not be generalizable to individuals with milder injuries who either do not present to medical care or who present to outpatient clinics, urgent cares, or lower-level trauma centers. Additionally, our analytic sample consisted of individuals with TBI that were able to complete cognitive testing at 2 weeks postinjury, most of whom had GCS 13 to 15; only 6.6% had GCS 3 to 12, so our results may also not be generalized to individuals with injuries of greater severity.

## Conclusions

In conclusion, the results of this study provide evidence that 2 weeks post-TBI global cognitive status is lower among individuals with diabetes, but that global cognitive trajectories in the first-year postinjury are similar among individuals with vs without comorbid vascular risk factors. This has important clinical implications for managing postinjury cognitive recovery expectations and return to activity timeline (eg, return to work) among patients with select vascular risk factors following TBI. As vascular risk factors, such as diabetes, are modifiable (eg, with improved glucose control), future work over longer follow-up and with consideration of postinjury changes in vascular risk factor burden is warranted.

## Notes

Supplement 1.
eMethods.
**eTable 1.** Fit of cross-sectional 1-factor models **eTable 2.** Fit of longitudinal invariance models **eTable 3.** Participant characteristics, stratified by number of vascular risk factors **eTable 4.** Comparison of characteristics between individuals with and without 1 year cognitive data (among individuals included in analyses) **eTable 5.** Associations of treated and untreated preinjury hypertension, diabetes, and hyperlipidemia with 2-week cognition and change in cognition from 2 weeks to 1 year postinjury **eTable 6. **Associations of preinjury vascular risk factors with 2 weeks cognition and change in cognition from 2 weeks to 1 year postinjury, adding age to model **eTable 7.** Associations of the number of preinjury vascular risk factors with 2-week cognition and change in cognition from 2 weeks to 1 year postinjury, adding age to model **eTable 8.** Unweighted complete case sensitivity analysis of associations of preinjury vascular risk factors with 2 weeks cognition and change in cognition from 2 weeks to 1 year postinjury **eTable 9. **Unweighted complete case sensitivity analysis of associations of the number of preinjury vascular risk factors with 2-week cognition and change in cognition from 2 weeks to 1 year postinjury **eFigure 1. **Structure and standardized loadings from strict longitudinal invariance model of the general cognitive factor **eFigure 2.** Participant flow diagram
eReferences.

Supplement 2.
Data Sharing Statement
