# Supplementary material for: Vascular Risk Factors and 1-Year Cognitive Change Among Individuals With Traumatic Brain Injury
Source: JAMA Netw Open. 2025 Aug 8;8(8):e2525719. doi: 10.1001/jamanetworkopen.2025.25719 (PMC12334961; doi:10.1001/jamanetworkopen.2025.25719)
Supplement: Supplement 1. — eMethods. eTable 1. Fit of cross-sectional 1-factor models eTable 2. Fit of longitudinal invariance models eTable 3. Participant characteristics, stratified by number of vascular risk factors eTable 4. Comparison of characteristics between individuals with and without 1 year cognitive data (among individuals included in analyses) eTable 5. Associations of treated and untreated preinjury hypertension, diabetes, and hyperlipidemia with 2-week cognition and change in cognition from 2 weeks to 1 year postinjury eTable 6. Associations of preinjury vascular risk factors with 2 weeks cognition and change in cognition from 2 weeks to 1 year postinjury, adding age to model eTable 7. Associations of the number of preinjury vascular risk factors with 2-week cognition and change in cognition from 2 weeks to 1 year postinjury, adding age to model eTable 8. Unweighted complete case sensitivity analysis of associations of preinjury vascular risk factors with 2 weeks cognition and change in cognition from 2 weeks to 1 year postinjury eTable 9. Unweighted complete case sensitivity analysis of associations of the number of preinjury vascular risk factors with 2-week cognition and change in cognition from 2 weeks to 1 year postinjury eFigure 1. Structure and standardized loadings from strict longitudinal invariance model of the general cognitive factor eFigure 2. Participant flow diagram eReferences. [file jamanetwopen-e2525719-s001.pdf]

Supplemental Online Content

Schneider ALC, Hunzinger KJ, Brett BL, et al; TRACK-TBI Study Investigators. Associations of preinjury vascular risk factors with 1-year cognitive change after traumatic brain injury: a TRACK-TBI study. *JAMA Netw Open*. 2025;8(8):e2525719.
doi:10.1001/jamanetworkopen.2025.25719

eMethods.
eTable 1. Fit of cross-sectional 1-factor models
eTable 2. Fit of longitudinal invariance models
eTable 3. Participant characteristics, stratified by number of vascular risk factors
eTable 4. Comparison of characteristics between individuals with and without 1-year cognitive data (among individuals included in analyses)
eTable 5. Associations of treated and untreated preinjury hypertension, diabetes, and hyperlipidemia with 2-week cognition and change in cognition from 2-weeks to 1-year postinjury
eTable 6. Associations of preinjury vascular risk factors with 2-weeks cognition and change in cognition from 2-weeks to 1-year postinjury, adding age to model
eTable 7. Associations of the number of preinjury vascular risk factors with 2-week cognition and change in cognition from 2-weeks to 1-year postinjury, adding age to model
eTable 8. Unweighted complete case sensitivity analysis of associations of preinjury vascular risk factors with 2-weeks cognition and change in cognition from 2-weeks to 1-year postinjury
eTable 9. Unweighted complete case sensitivity analysis of associations of the number of preinjury vascular risk factors with 2-week cognition and change in cognition from 2-weeks to 1-year postinjury
eFigure 1. Structure and standardized loadings from strict longitudinal invariance model of the general cognitive factor
eFigure 2. Participant flow diagram
eReferences.

This supplemental material has been provided by the authors to give readers additional information about their work.

© 2025 Schneider ALC, et al. *JAMA Network Open*.

eMethods

Hypertension, Diabetes, Hyperlipidemia, and Smoking Cessation Medications

Medications were assigned the following therapeutic classes using the Multum Lexicon classification system: (1) hypertension medications: aldosterone receptor antagonists, alpha blockers, angiotensin converting enzyme inhibitors, angiotensin receptor blockers, beta blockers, calcium channel blockers, centrally acting antihypertensives, direct vasodilators, diuretics, renin inhibitors, (2) diabetes medications: alpha-glucosidase inhibitors, amylin analogs, biguanides, dipeptidyl peptidase 4 inhibitors, glucose like peptide-1 receptor agonists, insulin, meglitinides, sodium-glucose co-transporter-2 inhibitors, sulfonylureas, thiazolidinediones, (3) hyperlipidemia medications: bile acid sequestrants, cholesterol absorption inhibitors, fibrates, statins, and (4) smoking cessation medications: bupropion, nicotine replacement therapy, varenicline.

Global Cognitive Factor Score

To create a global cognitive factor score, factor analyses were conducted with M*plus* version 8.5 using full information maximum likelihood estimation with robust standard errors. Full-information maximum likelihood estimation is considered robust to missing at random missingness mechanisms. We performed confirmatory factor analysis (CFA) to test the fit of a 1-factor model comprising the five cognitive outcomes: RAVLT trials 1–5 total score, RAVLT delayed recall score, TMT part A time, TMT part B time, and WAIS-IV PSI. One model was fit for each follow-up time-point, and correlated residuals were allowed between the two RAVLT items and between the two TMT items (**eFigure 1, eTable 1**). The literature considers a model to fit well if root mean square error of approximation (RMSEA) is <0.06 (acceptable if <0.08) and if comparative fit index (CFI) and Tucker-Lewis index (TLI) are >0.95.[1] We did not use χ^2

© 2025 Schneider ALC, et al. *JAMA Network Open*.

goodness-of-fit tests as they tend to be oversensitive to minor deviations in fit in large samples, and are therefore not recommended for evaluating model fit in large samples.[1]

Longitudinal measurement invariance modeling established the degree to which the global cognitive factor reflects the same construct from 2 weeks to 12 months post-injury. This was tested through a series of CFA models in which we imposed increasingly stringent constraints on the equivalence of model parameters between groups: (1) that the same items load on the same factors, allowing other parameters to vary between groups (configural invariance); (2) that, additionally, factor loadings are equivalent between groups (weak invariance); (3) that, additionally, item intercepts are equivalent between groups (strong invariance); and (4) that, additionally, residual variances are equivalent between groups (strict invariance). The fit of these models was adequate to consider the global cognitive factor reasonably invariant to time (**eTable 2**). The literature supports considering a model to be longitudinally invariant if delta RMSEA ≤0.015 and delta CFI is ≤0.10.[2,3] Estimated global cognitive factor scores were extracted from the strict invariance model for use in further analyses.

Covariates

A priori selected covariates in statistical models were ascertained at study enrollment and included the following: age (years, continuous), sex (female; male), race (Black; non-Black; ascertained from self-/proxy-report or medical records), ethnicity (Hispanic; non-Hispanic; ascertained from self-/proxy-report or medical records), education (years, continuous), TBI severity (Glasgow Coma Scale [GCS] 13-15 and acute head CT negative for intracranial findings; GCS 13-15 and acute head CT positive for intracranial findings; GCS 3-12), prior TBI (yes; no), and psychiatric history (yes; no).

© 2025 Schneider ALC, et al. *JAMA Network Open*.

eTable 1. Fit of cross-sectional 1-factor models

	χ^2	df	RMSEA (90% CI)	CFI	TLI
2-Weeks	18.00	3	0.05 (0.03, 0.07)	0.996	0.988
6-Months	3.98	3	0.01 (0.00, 0.04)	1.000	0.999
1-Year	7.74	3	0.03 (0.00, 0.06)	0.999	0.995

Abbreviations: CFI, Comparative Fit Index; RMSEA, root mean square error of approximation, TLI, Tucker-Lewis Index.

© 2025 Schneider ALC, et al. *JAMA Network Open*.

eTable 2. Fit of longitudinal invariance models

	-2LL	k	BIC	RMSEA	Δ RMSEA	CFI	Δ CFI
Configural	-95985	72	192528	.073	Reference	.951	Reference
Weak	-96041	64	192578	.071	-.002	.947	-.004
Strong	-96258	56	192952	.081	.010	.925	-.022
Strict	-96329	46	193015	.077	-.004	.923	-.002

Abbreviations: BIC, Bayesian Information Criterion; CFI, Comparative Fit Index; RMSEA, root mean square error of approximation.

© 2025 Schneider ALC, et al. *JAMA Network Open*.

eTable 3. Participant characteristics, stratified by number of vascular risk factors

	0 Vascular Risk Factors (n=689)	1 Vascular Risk Factor (n=473)	2+ Vascular Risk Factors (n=151)
Age (years), mean (SD)	34.9 (14.0)	38.2 (16.2)	57.7 (13.4)
Sex, n (%)			
Female	234 (34.0)	141 (29.8)	53 (35.1)
Male	455 (66.0)	332 (70.2)	98 (64.9)
Race, n (%)			
Black	94 (13.6)	111 (23.5)	22 (14.6)
White	535 (77.7)	341 (72.1)	119 (78.8)
Other*	60 (8.7)	21 (4.4)	10 (6.6)
Ethnicity, n (%)			
Hispanic	156 (22.6)	74 (15.6)	26 (17.2)
Non-Hispanic	533 (77.4)	399 (84.4)	125 (8.28)
Education (years), mean (SD)	13.9 (2.8)	13.3 (2.7)	13.6 (3.3)
TBI severity, n (%)			
GCS 13-15 and acute head CT negative for intracranial findings	449 (65.2)	296 (62.6)	77 (51.0)
GCS 13-15 and acute head CT positive for intracranial findings	186 (27.0)	149 (31.5)	69 (45.7)
GCS 3-12	54 (7.8)	28 (5.9)	5 (3.3)
Hypertension, n (%)	0	89 (18.8)	132 (87.4)
Treated hypertension, n (%)	0	72 (15.2)	108 (71.5)
Untreated hypertension, n (%)	0	17 (3.6)	24 (15.9)
Diabetes, n (%)	0	25 (5.3)	73 (48.3)
Treated diabetes, n (%)	0	14 (3.0)	58 (38.4)
Untreated diabetes, n (%)	0	11 (2.3)	15 (9.9)
Hyperlipidemia, n (%)	0	27 (5.7)	89 (58.9)
Treated hyperlipidemia, n (%)	0	18 (3.8)	74 (49.0)
Untreated hyperlipidemia, n (%)	0	9 (1.9)	15 (9.9)
Smoking, n (%)	0	332 (22.5)	61 (40.4)
Prior TBI, n (%)	138 (19.9)	129 (27.3)	25 (16.6)
Psychiatric history, n (%)	123 (17.9)	119 (25.2)	52 (34.4)

*Other race includes American Indian, Alaskan Native, Asian, Native Hawaiian/Pacific Islander, and Unknown.

© 2025 Schneider ALC, et al. *JAMA Network Open*.

eTable 4. Comparison of characteristics between individuals with and without 1-year cognitive data (among individuals included in analyses)

	Participants Included in Analyses (n=1,313)	
	Included in Analyses with 1-Year Cognitive Data (n=884)	Included in Analyses without 1-Year Cognitive Data (n=429)
Age (years), mean (SD)	39.8 (16.7)	36.5 (15.3)
Sex, n (%)		
Female	298 (33.7)	130 (30.3)
Male	586 (66.3)	299 (69.7)
Race, n (%)		
Black	154 (17.4)	73 (17.0)
White	660 (74.7)	335 (78.1)
Other*	70 (7.9)	21 (4.9)
Ethnicity, n (%)		
Hispanic	128 (14.5)	101 (23.5)
Non-Hispanic	694 (78.5)	328 (76.5)
Missing	0	0
Education (years), mean (SD)	14.0 (2.8)	13.0 (2.9)
TBI severity, n (%)		
GCS 13-15 and acute head CT negative for intracranial findings	553 (62.6)	269 (62.7)
GCS 13-15 and acute head CT positive for intracranial findings	286 (32.4)	118 (27.5)
GCS 3-12	45 (5.1)	42 (9.8)
Missing	0	0
Hypertension, n (%)	159 (18.0)	62 (14.5)
Treated hypertension, n (%)	131 (14.8)	49 (11.4)
Untreated hypertension, n (%)	28 (3.2)	13 (3.0)
Diabetes, n (%)	74 (8.4)	34 (7.9)
Treated diabetes, n (%)	45 (5.1)	27 (6.3)
Untreated diabetes, n (%)	19 (2.2)	7 (1.6)
Hyperlipidemia, n (%)	92 (10.4)	24 (5.6)
Treated hyperlipidemia, n (%)	74 (8.4)	18 (4.2)
Untreated hyperlipidemia, n (%)	18 (2.0)	6 (1.4)
Smoking, n (%)	92 (10.4)	137 (31.9)
Prior TBI, n (%)	190 (21.5)	101 (23.5)
Psychiatric history, n (%)	214 (24.2)	80 (18.7)

*Other race includes American Indian, Alaskan Native, Asian, Native Hawaiian/Pacific Islander, and Unknown.

© 2025 Schneider ALC, et al. *JAMA Network Open*.

eTable 5. Associations of treated and untreated pre-injury hypertension, diabetes, and hyperlipidemia with 2-week cognition and change in cognition from 2-weeks to 1-year post-injury

	Treated hypertension versus no hypertension	Untreated hypertension versus no hypertension	Treated diabetes versus no diabetes	Untreated diabetes versus no diabetes	Treated hyperlipidemia versus no hyperlipidemia	Untreated hyperlipidemia versus no hyperlipidemia
**Global cognitive factor z-score**						
Mean Difference at 2-weeks, (95% CI)	-0.02 (-0.16, 0.11)	-0.20 (-0.43, 0.04)	**-0.32 (-0.57, -0.07)**	-0.06 (-0.33, 0.21)	-0.17 (-0.37, 0.02)	0.13 (-0.13, 0.40)
Mean Difference in change from 2-weeks to 1-year, (95% CI)	-0.03 (-0.18, 0.12)	0.17 (-0.05, 0.40)	**-0.29 (-0.57, -0.02)**	0.08 (-0.20, 0.35)	0.10 (-0.06, 0.26)	0.09 (-0.11, 0.28)
**RAVLT trials 1–5 total (immediate recall) z-score**						
Mean Difference at 2-weeks, (95% CI)	-0.09 (-0.26, 0.08)	-0.21 (-0.47, 0.06)	-0.09 (-0.35, 0.17)	0.05 (-0.19, 0.29)	**-0.23 (-0.43, -0.03)**	0.27 (-0.02, 0.56)
Mean Difference in change from 2-weeks to 1-year, (95% CI)	-0.18 (-0.37, 0.02)	0.10 (-0.24, 0.43)	-0.26 (-0.53, 0.01)	-0.03 (-0.44, 0.38)	-0.06 (-0.27, 0.16)	-0.06 (-0.47, 0.36)
**RAVLT delayed recall z-score**						
Mean Difference at 2-weeks, (95% CI)	-0.08 (-0.26, 0.10)	-0.14 (-0.43, 0.14)	0.03 (-0.23, 0.29)	0.11 (-0.27, 0.48)	-0.11 (-0.32, 0.10)	0.26 (-0.18, 0.70)
Mean Difference in change from 2-weeks to 1-year, (95% CI)	-0.16 (-0.37, 0.06)	0.07 (-0.24, 0.38)	-0.22 (-0.50, 0.06)	-0.04 (-0.52, 0.45)	-0.12 (-0.34, 0.11)	0.09 (-0.41, 0.59)
**TMT part A z-score**						
Mean Difference at 2-weeks, (95% CI)	0.04 (-0.14, 0.21)	0.20 (-0.12, 0.52)	**0.36 (0.05, 0.67)**	0.07 (-0.32, 0.47)	0.12 (-0.12, 0.36)	-0.11 (-0.40, 0.19)
Mean Difference in change from 2-weeks to 1-year, (95% CI)	0.01 (-0.21, 0.24)	-0.21 (-0.53, 0.10)	0.26 (-0.18, 0.70)	-0.29 (-0.64, 0.06)	-0.11 (-0.37, 0.14)	-0.05 (-0.31, 0.21)
**TMT part B z-score**						
Mean Difference at 2-weeks, (95% CI)	-0.05 (-0.22, 0.12)	0.17 (-0.14, 0.47)	**0.37 (0.04, 0.69)**	0.12 (-0.25, 0.48)	0.17 (-0.07, 0.41)	-0.18 (-0.52, 0.16)
Mean Difference in change from 2-weeks to 1-year, (95% CI)	-0.01 (-0.25, 0.23)	-0.14 (-0.50, 0.22)	**0.53 (0.07, 0.99)**	-0.03 (-0.40, 0.33)	**-0.28 (-0.55, -0.01)**	0.04 (-0.29, 0.37)
**WAIS-IV PSI z-score**						
Mean Difference at 2-weeks, (95% CI)	0.04 (-0.13, 0.20)	-0.25 (-0.52, 0.03)	-0.23 (-0.46, 0.00)	-0.06 (-0.37, 0.26)	-0.17 (-0.38, 0.04)	-0.03 (-0.31, 0.25)
Mean Difference in change from 2-weeks to 1-year, (95% CI)	-0.09 (-0.27, 0.09)	0.11 (-0.16, 0.38)	**-0.29 (-0.56, -0.03)**	-0.19 (-0.66, 0.28)	0.03 (-0.16, 0.22)	0.18 (-0.12, 0.48)

© 2025 Schneider ALC, et al. *JAMA Network Open*.

IPA weights-adjusted GEE models were performed to assess the association of each individual vascular risk factor with each cognitive outcome. Each model included individual vascular risk factor, time-points, age, sex, race, ethnicity, years of education, TBI severity, prior TBI and psychiatric history, and the following interactions (vascular risk factor x time-points, age x time-points, sex x time-points, years of education x time-points, TBI severity x time-points).

Note: Bold text indicates $p<0.05$.

© 2025 Schneider ALC, et al. *JAMA Network Open*.

eTable 6. Associations of pre-injury vascular risk factors with 2-weeks cognition and change in cognition from 2-weeks to 1-year post-injury, adding age^2 to model

	Hypertension versus no hypertension	Diabetes versus no diabetes	Hyperlipidemia versus no hyperlipidemia	Smoking versus no smoking
**Global cognitive factor z-score**				
Mean Difference at 2-weeks, (95% CI)	-0.03 (-0.15, 0.10)	**-0.23 (-0.43, -0.03)**	-0.06 (-0.22, 0.01)	**-0.08 (-0.16, 0.00)**
Mean Difference in change from 2-weeks to 1-year, (95% CI)	0.01 (-0.13, 0.14)	-0.18 (-0.40, 0.04)	0.09 (-0.04, 0.22)	-0.01 (-0.10, 0.08)
**RAVLT trials 1–5 total (immediate recall) z-score**				
Mean Difference at 2-weeks, (95% CI)	-0.07 (-0.23, 0.08)	-0.03 (-0.23, 0.17)	-0.06 (-0.24, 0.12)	-0.10 (-0.21, 0.01)
Mean Difference in change from 2-weeks to 1-year, (95% CI)	-0.12 (-0.31, 0.06)	-0.19 (-0.42, 0.05)	-0.06 (-0.26, 0.14)	-0.11 (-0.25, 0.04)
**RAVLT delayed recall z-score**				
Mean Difference at 2-weeks, (95% CI)	-0.07 (-0.23, 0.10)	0.07 (-0.15, 0.29)	0.01 (-0.19, 0.21)	-0.06 (-0.18, 0.06)
Mean Difference in change from 2-weeks to 1-year, (95% CI)	-0.11 (-0.30, 0.08)	-0.17 (-0.41, 0.08)	-0.07 (-0.29, 0.14)	-0.12 (-0.26, 0.03)
**TMT part A z-score**				
Mean Difference at 2-weeks, (95% CI)	0.02 (-0.14, 0.18)	0.25 (-0.01, 0.51)	0.00 (-0.21, 0.20)	0.06 (-0.05, 0.17)
Mean Difference in change from 2-weeks to 1-year, (95% CI)	-0.03 (-0.22, 0.17)	0.10 (-0.24, 0.44)	-0.10 (-0.30, 0.11)	0.02 (-0.10, 0.15)
**TMT part B z-score**				
Mean Difference at 2-weeks, (95% CI)	-0.05 (-0.20, 0.11)	**0.27 (0.01, 0.53)**	0.03 (-0.17, 0.24)	0.08 (-0.02, 0.18)
Mean Difference in change from 2-weeks to 1-year, (95% CI)	-0.03 (-0.25, 0.18)	0.36 (-0.01, 0.73)	-0.20 (-0.43, 0.02)	0.00 (-0.13, 0.13)
**WAIS-IV PSI z-score**				
Mean Difference at 2-weeks, (95% CI)	-0.04 (-0.19, 0.11)	**-0.20 (-0.39, 0.00)**	-0.18 (-0.35, 0.00)	-0.06 (-0.16, 0.05)
Mean Difference in change from 2-weeks to 1-year, (95% CI)	-0.05 (-0.21, 0.11)	**-0.26 (-0.49, -0.02)**	0.06 (-0.10, 0.23)	-0.04 (-0.16, 0.09)

IPA weights-djusted GEE models were performed to assess the association of each individual vascular risk factor with each cognitive outcome. Each model included individual vascular risk factor, time-points, age, age^2, sex, race, ethnicity, years of education, TBI severity, prior TBI and psychiatric history, and the following interactions (vascular risk factor x time-points, age x time-points, age^2 x time-points, sex x time-points, years of education x time-points, TBI severity x time-points).

Note: Bold text indicates p<0.05.

© 2025 Schneider ALC, et al. *JAMA Network Open*.

eTable 7. Associations of the number of pre-injury vascular risk factors with 2-week cognition and change in cognition from 2-weeks to 1-year post-injury, adding age^2 to model

	1 versus 0 vascular risk factor	2+ versus 0 vascular risk factors
**Global cognitive factor z-score**		
Mean Difference at 2-weeks, (95% CI)	-0.02 (-0.10, 0.06)	**-0.18 (-0.32, -0.04)**
Mean Difference in change from 2-weeks to 1-year, (95% CI)	-0.02 (-0.10, 0.07)	-0.05 (-0.21, 0.11)
**RAVLT trials 1–5 total (immediate recall) z-score**		
Mean Difference at 2-weeks, (95% CI)	-0.10 (-0.20, 0.01)	-0.14 (-0.32, 0.04)
Mean Difference in change from 2-weeks to 1-year, (95% CI)	-0.12 (-0.26, 0.02)	-0.18 (-0.39, 0.03)
**RAVLT delayed recall z-score**		
Mean Difference at 2-weeks, (95% CI)	-0.05 (-0.16, 0.07)	-0.05 (-0.24, 0.14)
Mean Difference in change from 2-weeks to 1-year, (95% CI)	**-0.16 (-0.30, -0.02)**	-0.17 (-0.39, 0.05)
**TMT part A z-score**		
Mean Difference at 2-weeks, (95% CI)	-0.01 (-0.12, 0.09)	0.18 (-0.02, 0.38)
Mean Difference in change from 2-weeks to 1-year, (95% CI)	0.04 (-0.08, 0.15)	0.02 (-0.23, 0.26)
**TMT part B z-score**		
Mean Difference at 2-weeks, (95% CI)	0.02 (-0.08, 0.12)	0.12 (-0.07, 0.31)
Mean Difference in change from 2-weeks to 1-year, (95% CI)	-0.01 (-0.13, 0.12)	0.04 (-0.21, 0.29)
**WAIS-PSI z-score**		
Mean Difference at 2-weeks, (95% CI)	0.04 (-0.06, 0.15)	**-0.24 (-0.41, -0.07)**
Mean Difference in change from 2-weeks to 1-year, (95% CI)	-0.05 (-0.17, 0.07)	-0.09 (-0.27, 0.09)

IPA weights-djusted GEE models were performed to assess the association of number of vascular risk factors with each cognitive outcome. Each model included number of vascular risk factors, time-points, age, age^2, sex, race, ethnicity, years of education, TBI severity, prior TBI, and psychiatric history, and the following interactions (number of vascular risk factors x time-points, age x time-points, age^2 x time-points, sex x time-points, years of education x time-points, TBI severity x time-points).

Note: Bold text indicates $p<0.05$.

© 2025 Schneider ALC, et al. *JAMA Network Open*.

eTable 8. Unweighted complete case sensitivity analysis of associations of pre-injury vascular risk factors with 2-weeks cognition and change in cognition from 2-weeks to 1-year post-injury

	Hypertension versus no hypertension	Diabetes versus no diabetes	Hyperlipidemia versus no hyperlipidemia	Smoking versus no smoking
**Global cognitive factor z-score**				
Mean Difference at 2-weeks, (95% CI)	-0.09 (-0.22, 0.04)	**-0.23 (-0.43, -0.03)**	-0.10 (-0.26, 0.06)	-0.06 (-0.14, 0.03)
Mean Difference in change from 2-weeks to 1-year, (95% CI)	-0.01 (-0.07, 0.04)	-0.07 (-0.14, 0.00)	0.01 (-0.04, 0.07)	-0.03 (-0.07, 0.00)
**RAVLT trials 1–5 total (immediate recall) z-score**				
Mean Difference at 2-weeks, (95% CI)	-0.13 (-0.28, 0.02)	-0.05 (-0.25, 0.14)	-0.11 (-0.28, 0.07)	-0.11 (-0.22, 0.00)
Mean Difference in change from 2-weeks to 1-year, (95% CI)	-0.06 (-0.21, 0.01)	-0.14 (-0.34, 0.05)	-0.04 (-0.21, 0.13)	-0.07 (-0.19, 0.05)
**RAVLT delayed recall z-score**				
Mean Difference at 2-weeks, (95% CI)	-0.08 (-0.25, 0.08)	0.05 (-0.16, 0.27)	-0.02 (-0.21, 0.18)	-0.07 (-0.18, 0.05)
Mean Difference in change from 2-weeks to 1-year, (95% CI)	-0.11 (-0.28, 0.07)	-0.11 (-0.32, 0.10)	-0.10 (-0.29, 0.10)	-0.07 (-0.20, 0.05)
**TMT part A z-score**				
Mean Difference at 2-weeks, (95% CI)	0.08 (-0.08, 0.24)	0.23 (-0.02, 0.48)	0.04 (-0.16, 0.24)	0.03 (-0.08, 0.13)
Mean Difference in change from 2-weeks to 1-year, (95% CI)	-0.01 (-0.15, 0.13)	-0.05 (-0.25, 0.16)	-0.01 (-0.18, 0.16)	0.06 (-0.04, 0.16)
**TMT part B z-score**				
Mean Difference at 2-weeks, (95% CI)	0.03 (-0.13, 0.19)	0.24 (-0.01, 0.49)	0.09 (-0.11, 0.29)	0.04 (-0.06, 0.14)
Mean Difference in change from 2-weeks to 1-year, (95% CI)	0.03 (-0.11, 0.17)	0.17 (-0.03, 0.37)	-0.10 (-0.26, 0.06)	0.04 (-0.06, 0.12)
**WAIS-IV PSI z-score**				
Mean Difference at 2-weeks, (95% CI)	-0.06 (-0.02, 0.09)	**-0.20 (-0.39, -0.01)**	-0.14 (-0.31, 0.04)	-0.03 (-0.14, 0.07)
Mean Difference in change from 2-weeks to 1-year, (95% CI)	-0.03 (-0.15, 0.09)	**-0.20 (-0.38, -0.02)**	0.01 (-0.12, 0.14)	-0.09 (-0.18, 0.01)

Adjusted GEE models were performed to assess the association of each individual vascular risk factor with each cognitive outcome. Each model included individual vascular risk factor, time-points, age, sex, race, ethnicity, years of education, TBI severity, prior TBI and psychiatric history, and the following interactions (vascular risk factor x time-points, age x time-points, sex x time-points, years of education x time-points, TBI severity x time-points).

Note: Bold text indicates p<0.05.

© 2025 Schneider ALC, et al. *JAMA Network Open*.

eTable 9. Unweighted complete case sensitivity analysis of associations of the number of pre-injury vascular risk factors with 2-week cognition and change in cognition from 2-weeks to 1-year post-injury

	1 versus 0 vascular risk factor	2+ versus 0 vascular risk factors
**Global cognitive factor z-score**		
Mean Difference at 2-weeks, (95% CI)	-0.03 (-0.11, 0.05)	**-0.19 (-0.34, -0.03)**
Mean Difference in change from 2-weeks to 1-year, (95% CI)	-0.03 (-0.07, 0.00)	-0.04 (-0.10, 0.02)
**RAVLT trials 1–5 total (immediate recall) z-score**		
Mean Difference at 2-weeks, (95% CI)	**-0.13 (-0.24, -0.02)**	**-0.19 (-0.36, -0.02)**
Mean Difference in change from 2-weeks to 1-year, (95% CI)	-0.08 (-0.19, 0.04)	-0.10 (-0.27, 0.08)
**RAVLT delayed recall z-score**		
Mean Difference at 2-weeks, (95% CI)	-0.07 (-0.19, 0.04)	-0.08 (-0.26, 0.11)
Mean Difference in change from 2-weeks to 1-year, (95% CI)	**-0.13 (-0.25, -0.01)**	-0.12 (-0.31, 0.07)
**TMT part A z-score**		
Mean Difference at 2-weeks, (95% CI)	-0.01 (-0.11, 0.10)	0.17 (-0.03, 0.36)
Mean Difference in change from 2-weeks to 1-year, (95% CI)	0.08 (-0.02, 0.17)	-0.01 (-0.18, 0.16)
**TMT part B z-score**		
Mean Difference at 2-weeks, (95% CI)	0.03 (-0.07, 0.13)	0.12 (-0.07, 0.31)
Mean Difference in change from 2-weeks to 1-year, (95% CI)	-0.01 (-0.09, 0.08)	0.06 (-0.10, 0.22)
**WAIS-PSI z-score**		
Mean Difference at 2-weeks, (95% CI)	0.05 (-0.05, 0.16)	**-0.21 (-0.38, -0.05)**
Mean Difference in change from 2-weeks to 1-year, (95% CI)	-0.08 (-0.17, 0.02)	-0.12 (-0.26, 0.01)

Adjusted GEE models were performed to assess the association of number of vascular risk factors with each cognitive outcome. Each model included number of vascular risk factors, time-points, age, sex, race, ethnicity, years of education, TBI severity, prior TBI, and psychiatric history, and the following interactions (number of vascular risk factors x time-points, age x time-points, sex x time-points, years of education x time-points, TBI severity x time-points).

Note: Bold text indicates p<0.05.

© 2025 Schneider ALC, et al. *JAMA Network Open*.

eFigure 1. Structure and standardized loadings from strict longitudinal invariance model of the general cognitive factor

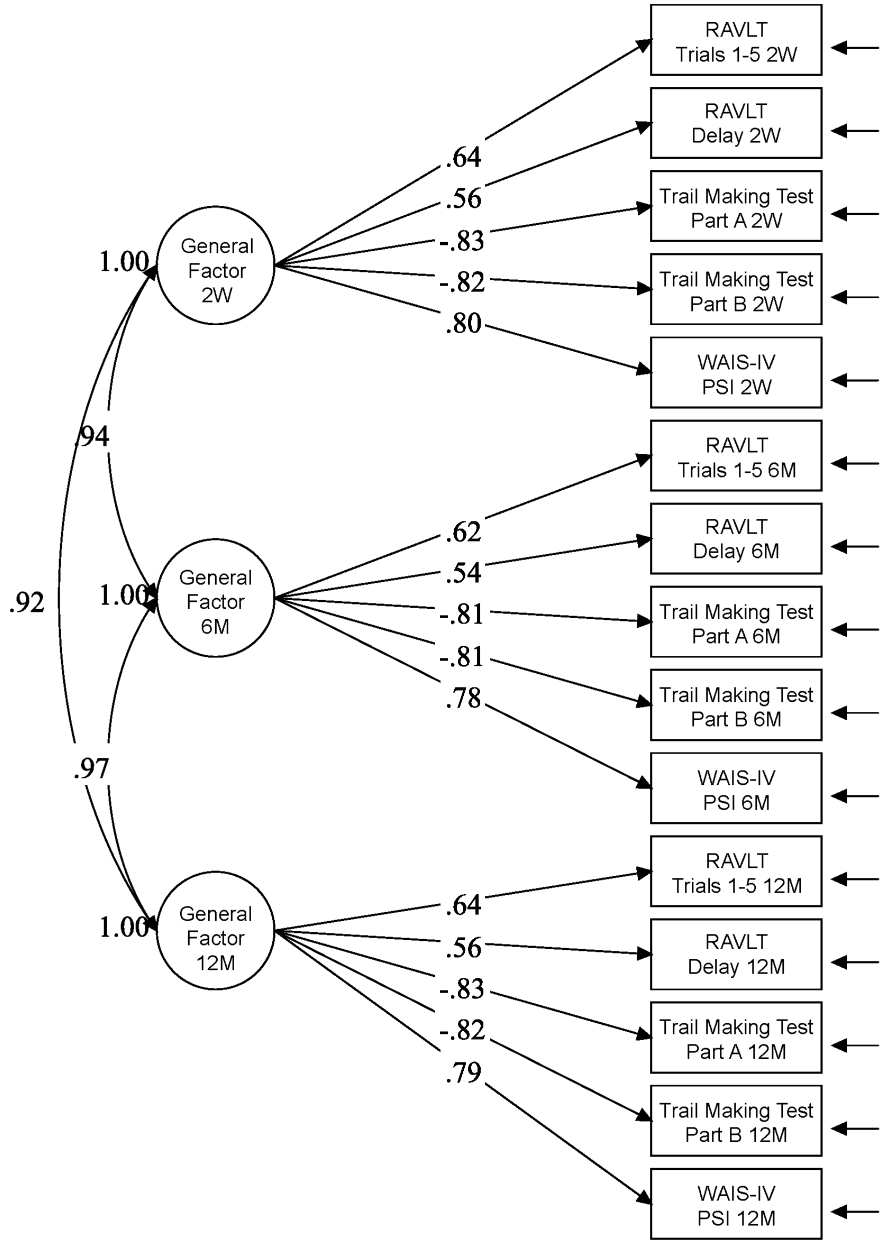

Residual correlations (suppressed in the figure for readability) were allowed to be non-zero between all RAVLT items within and across time; and between all Trail Making Test items within and across time.

© 2025 Schneider ALC, et al. *JAMA Network Open*.

eFigure 2. Participant flow diagram

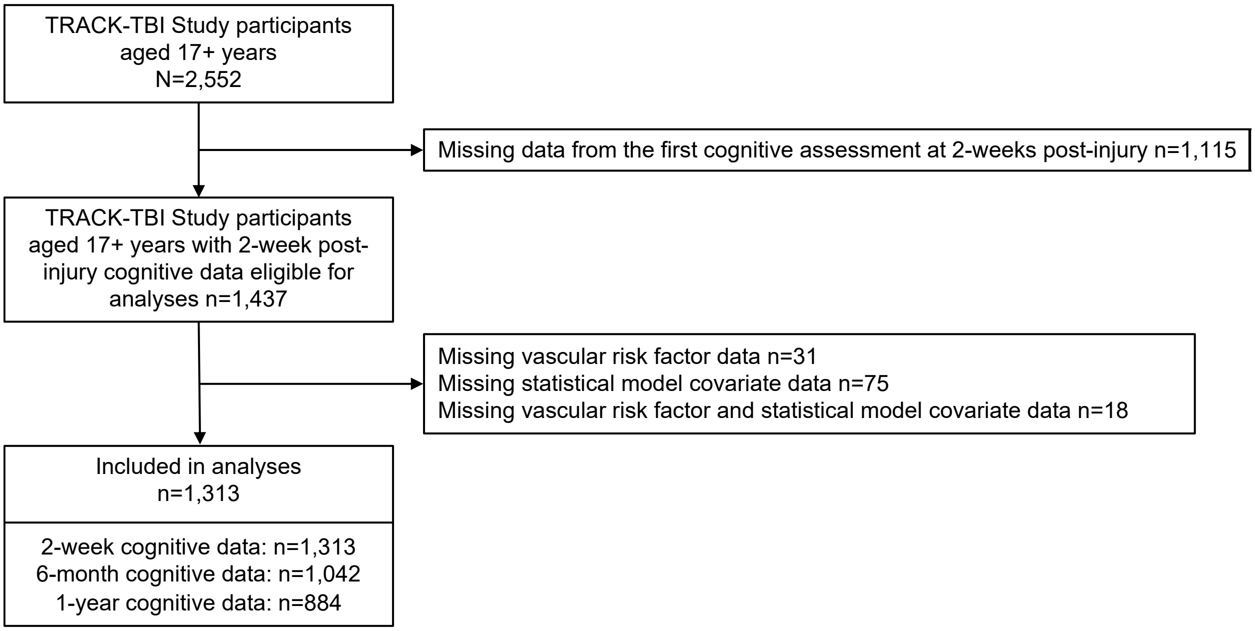

© 2025 Schneider ALC, et al. *JAMA Network Open*.

eReferences.

1. Hu L, Bentler P. Cutoff criteria for fit indices in covariance structure analysis: Conventional crtieria versus new alternatives. *Structural Equation Modeling*. 1999;6:1-55.

2. Chen FF. Sensitivity of goodness of fit indexes to lack of measurement invariance. *Structural Equation Modeling*. 2007;14(3):464-504.

3. Cheung GW, Rensvold RB. Evaluating goodness-of-fit indexes for testing measurement invariance. *Structural Equation Modeling*. 2002;9(2):233-255.

© 2025 Schneider ALC, et al. *JAMA Network Open*.
