# Supplementary material for: Vascular Risk Factors and 1-Year Cognitive Change Among Individuals With Traumatic Brain Injury
Source: JAMA Netw Open. 2025 Aug 8;8(8):e2525719. doi: 10.1001/jamanetworkopen.2025.25719 (PMC12334961; doi:10.1001/jamanetworkopen.2025.25719)
Supplement: Supplement 2. — Data Sharing Statement [file jamanetwopen-e2525719-s002.pdf]

Data Sharing Statement

Data

Data available: Yes
Data types: Deidentified participant data, Data dictionary
How to access data: The TRACK-TBI Study data is available on FITBIR (https://fitbir.nih.gov).
When available: With publication

Supporting Documents

Document types: None

Additional Information

Who can access the data: The TRACK-TBI Study data is available on FITBIR (https://fitbir.nih.gov).
Types of analyses: The TRACK-TBI Study data is available on FITBIR (https://fitbir.nih.gov).
Mechanisms of data availability: The TRACK-TBI Study data is available on FITBIR (https://fitbir.nih.gov).
